# Novel Approaches for the Treatment of Post-Traumatic Stress Disorder: A Systematic Review of Non-Invasive Brain Stimulation Interventions and Insights from Clinical Trials

**DOI:** 10.3390/brainsci14030210

**Published:** 2024-02-24

**Authors:** Daniele Saccenti, Leandro Lodi, Andrea Stefano Moro, Simona Scaini, Barbara Forresi, Jacopo Lamanna, Mattia Ferro

**Affiliations:** 1 Department of Psychology, Sigmund Freud University, 20143 Milan, Italy; saccenti.phd@milano-sfu.it (D.S.); leo.lodi18@yahoo.com (L.L.); moro.phd@milano-sfu.it (A.S.M.); b.forresi@milano-sfu.it (B.F.); m.ferro@milano-sfu.it (M.F.); 2 Center for Behavioral Neuroscience and Communication (BNC), Vita-Salute San Raffaele University, 20132 Milan, Italy; 3 Child and Youth Lab, Sigmund Freud University of Milan, Via Ripa di Porta Ticinese 77, 20143 Milan, Italy; 4 Faculty of Psychology, Vita-Salute San Raffaele University, 20132 Milan, Italy

**Keywords:** post-traumatic stress disorder, PTSD, non-invasive brain stimulation, NIBS, transcranial magnetic stimulation, TMS, transcranial direct-current stimulation, tDCS, psychotherapy

## Abstract

First-line treatments for post-traumatic stress disorder (PTSD) encompass a wide range of pharmacotherapies and psychotherapies. However, many patients fail to respond to such interventions, highlighting the need for novel approaches. Due to its ability to modulate cortical activity, non-invasive brain stimulation (NIBS) could represent a valuable therapeutic tool. Therefore, the aim of this systematic review is to summarize and discuss the existing evidence on the ameliorative effects of NIBS on PTSD and comorbid anxiety and depressive symptoms. Our goal is also to debate the effectiveness of an integrated approach characterized by the combination of NIBS and psychotherapy. This search was conducted following the preferred reporting items for systematic reviews and meta-analyses (PRISMA) guidelines in the PubMed, PsycINFO, PsycARTICLES, PSYINDEX, MEDLINE, and ERIC databases. Overall, 31 studies met the eligibility criteria, yielding a total of 26 clinical trials employing transcranial magnetic stimulation (TMS) and 5 making use of transcranial direct-current stimulation (tDCS). From these studies, it emerged that NIBS consistently reduced overall PTSD symptoms’ severity as well as comorbid anxiety and depressive symptoms. Moreover, we speculate that combining NIBS with prolonged exposure or cognitive processing therapy might represent a promising therapeutic approach for consistently ameliorating subjects’ clinical conditions.

## 1. Introduction

Post-traumatic stress disorder (PTSD) is a debilitating psychiatric condition induced by exposure to a traumatic or stressful event(s), such as combat, accidents, assaults, terror attacks, and natural disasters, that is characterized by four main symptom clusters: (1) intrusive re-experiencing of the traumatic event through nightmares, flashbacks, or intrusive thoughts; (2) avoidance of trauma-related stimuli; (3) negative alterations in cognition and mood; and (4) hyperarousal symptoms, including exaggerated startle responses, anger outbursts, disturbed sleep, and sustained preparedness for alarm responses [1]. PTSD is often diagnosed along with other mental disorders, such as major depressive disorder (MDD), anxiety disorders, and substance use disorders (SUD) [2,3]. The lifetime prevalence of PTSD ranges from 3.9% to 7.8% among the general population and spikes up to a minimum of 9.4% if only military personnel are considered [4,5,6,7,8,9].

Evidence-based therapy for PTSD encompasses psychotherapy and pharmacotherapy [10,11,12]. To date, most of the empirical literature on psychotherapies for PTSD has focused on cognitive–behavioral therapy (CBT; [13]), with prolonged exposure (PE) therapy and eye movement desensitization and reprocessing (EMDR) representing the most widely evaluated and disseminated approaches [14,15]. However, PTSD is still difficult to treat: in fact, following intervention, two-thirds of patients remain fully or partially symptomatic [16]. Indeed, the enduring effects of current psychotherapeutic treatments last for 6 to 20 months at most [17,18]. Especially for those patients who fail to respond to first-line therapies or experience intolerable adverse effects, the development of novel treatment strategies that rapidly and effectively produce reductions in PTSD symptoms’ severity and guarantee the long-term maintenance of such improvements is, therefore, needed. In this framework, non-invasive brain stimulation (NIBS) techniques are receiving particular attention due to their ability to modulate cortical activity without causing any relevant side effects [19].

Neuromodulation represents a valid alternative to conventional therapeutic approaches, since PTSD is associated with disruptions in the neuronal networks involved in stress–fear response regulation. The fronto-limbic network, which connects the amygdala and hippocampus to many areas of the prefrontal cortex (PFC), including the ventromedial prefrontal cortex (vmPFC) and the anterior cingulate cortex (ACC) [20], is likely one of the most comprised [21,22]. Normally, the vmPFC assumes a regulatory function in modulating the stress-fear response by exerting control over the amygdala [23], which, in turn, is responsible for the perception and processing of emotions and emotional memories, playing a pivotal role in fear conditioning and extinction [24]. Among patients with PTSD, the vmPFC manifests hypoactivity and is no longer able to inhibit the amygdala [25], thus rendering it hyperactive, whereas the hippocampus exhibits hypoactivity, contributing to difficulties with fear extinction learning and contextualization of intrusive fear memories [26,27,28]. It has been hypothesized that such aberrant activity in fronto-limbic areas underlies core symptoms of PTSD including hyperarousal, negative alterations in cognition and mood, and intrusive memories [29,30,31]. Besides the fronto-limbic network, the default-mode (DMN) and salience (SN) networks have also been found to be altered in PTSD [32]. Disruptions in the DMN contribute to emotion dysregulation, dissociation, rumination, and numbing [33], whereas aberrant activity in the SN correlates with hyperarousal and a reduced capacity to discriminate between threatening and non-threatening stimuli [34].

Neuromodulation might heal these abnormalities and restore the physiological activation of these networks [35], thus decreasing patients’ PTSD symptomatology via different stimulation techniques, including transcranial magnetic stimulation (TMS) and transcranial electrical stimulation (TES). Specifically, TMS directly and focally modulates the brain activity of specific areas by inducing rapid and repeated changes in the electromagnetic field through the application of a copper coil over the scalp [36]. This tool has been consistently employed in the field of psychiatry, showing reasonable efficacy in treating MDD [37,38], SUD [39], and obsessive compulsive disorder [40]. Contrary to TMS, TES does not directly evoke action potentials in cortical neurons but rather endurably modulates spontaneous cortical activity and neuronal excitability through the application of a continuous (direct) electrical current, presumably reaching the target areas, using electrodes placed on the scalp [41]. Also, TES comprises a number of different techniques based on the stimulation protocol applied, i.e., transcranial direct-current stimulation (tDCS), alternating-current stimulation (tACS), and random noise stimulation (tRNS), and has been relatively less tested among clinical populations compared to TMS [42].

Several studies have been conducted in the last two decades on the therapeutic effects of NIBS on PTSD, which have been already systematized in recently published reviews and meta-analyses (e.g., [43,44,45]). However, only a few of these focused on the effects of an integrated combination of NIBS with other non-pharmacological evidence-based interventions. Indeed, the potentiation of PE with TMS could enhance the process of desensitization involved in fear extinction [46], thus leading to better treatment efficiency in patients with PTSD. To our knowledge, Kan et al. [44] were the only researchers who summarized the augmentation effects of TMS, highlighting that both mono- as well as integrated therapy yielded a significant reduction in participants’ PTSD symptoms’ severity. Yet, no specifications concerning the psychopharmacological and/or psychotherapeutic treatment that the subjects underwent during the study period were reported. Therefore, the aim of this systematic review is to (1) provide and discuss an updated collection of the existing evidence on the therapeutic effects of NIBS on the core symptoms of PTSD, (2) summarize the existing evidence on the therapeutic effects of NIBS on PTSD comorbid disorders such as anxiety and depression, and (3) discuss the combined efficacy of a potential new integrated treatment strategy.

## 2. Materials and Methods

The present review was carried out using the Preferred Reporting Items for Systematic reviews and Meta-Analyses (PRISMA) guidelines to ensure the systematicity and replicability of the obtained results [47]. No PRISMA registration has been carried out for this work.

### 2.1. Search Formula

To find relevant materials, the following electronic databases were used: PubMed and EBSCOhost, which comprises records retrieved from APA PsycINFO, APA PsycARTICLES, PSYNDEX: Literature and Tests, MEDLINE, and ERIC. The last search was performed on the 20th of January 2024, entering terms such as “non-invasive brain stimulation”, “NIBS”, “transcranial magnetic stimulation”, “TMS”, “transcranial direct current stimulation”, “tDCS”, “post-traumatic stress disorder”, and “PTSD”. In particular, the following search formula was entered in both databases: ((NIBS) OR (Non-invasive brain stimulation) OR (TMS) OR (Transcranial Magnetic Stimulation) OR (cTBS) OR (continuous theta burst stimulation) OR (iTBS) OR (intermittent theta burst stimulation) OR (tES) OR (transcranial electrical stimulation) OR (tDCS) OR (transcranial direct current stimulation) OR (tACS) OR (transcranial alternating current stimulation) OR (tRNS) OR (transcranial random noise stimulation)) AND ((PTSD) OR (post traumatic stress disorder) OR (posttraumatic stress disorder) OR (post-traumatic stress disorder)) AND ((clinical trial) OR ((open trial) OR (open label trial)) OR ((randomized controlled trial) OR (RCT))). Additional material was identified through manual selection.

### 2.2. Eligibility Criteria

Concerning the eligibility of studies relevant to understanding the effects of NIBS on PTSD, the PICOS (participants, intervention, comparison, outcome measure, and study design) method was applied [48]. To be included in this systematic review, studies needed to meet the following inclusion criteria:

- Participants: Studies conducted on adult participants (i.e., of 18 years of age or older) with a primary diagnosis of PTSD according to *Diagnostic and Statistical Manual of Mental Disorders, Fourth Edition* (DSM-IV), *Diagnostic and Statistical Manual of Mental Disorders, Fourth Edition, Text Revision* (DSM-IV-TR), *Diagnostic and Statistical Manual of Mental Disorders, Fifth Edition* (DSM-5), or *Diagnostic and Statistical Manual of Mental Disorders, Fifth Edition, Text Revision* (DSM-5-TR; [1]) classifications. Studies in which participants reported highly severe PTSD symptoms, i.e., a total score on the Post-Traumatic Stress Disorder Checklist—Civilian Version (PCL-C) greater than 50, were also included.
- Intervention: Studies in which NIBS was administered as a mono-therapy or combined with other evidence-based and non-surgical interventions, e.g., psychotherapy. Both TMS and TES, including tDCS, tACS, and tRNS, were considered to be eligible treatments.
- Comparison: The presence of at least one control group or condition that could be treated with a sham, i.e., inactive, stimulation, or active stimulation over a control site, e.g., Vertex, was not mandatory for inclusion.
- Outcome measure: Studies that evaluated potential reductions in participants’ PTSD symptoms’ severity according to standardized instruments. Overall PTSD symptoms’ severity was considered as the primary outcome, whereas general anxiety and depressive symptoms’ severity, assessed through standardized instruments, were considered secondary outcomes.
- Study Design: All types of quantitative clinical trials were included, as long as they met the above-mentioned inclusion criteria. Specifically, retrospective, controlled, or open-label studies with or without randomization were accepted if they provided at least one pre- and one post-treatment assessment of participants’ symptomatology.

Studies were excluded from the systematic review in the following cases:

- The sample included children, adolescents (i.e., of 16 years of age or below), adults with a secondary diagnosis of PTSD, or animal models.
- Stimulation was delivered invasively, e.g., through the implantation of electrodes in the brain, or coupled with other surgical procedures.
- PTSD symptoms’ severity was not assessed or compared among groups or conditions.
- They were non-original research studies (e.g., secondary sources, opinion-based, editorials, policy reviews and statements, commentaries), Master-level dissertations, conference presentations, conference proceedings where full-length articles were not available, preprints, single-case studies, correlational studies, narrative articles or reviews, or meta-analyses.
- Papers written in a language other than English were also excluded.

### 2.3. Study Screening and Selection Process

The first author ran the searches in the electronic databases, identified relevant studies, and removed duplicates using Zotero [49]. The second and the first author independently screened all the records following the inclusion/exclusion criteria. The screening process consisted of two stages: (1) authors detected whether non-original research articles, dissertations, conference presentations, proceedings, or preprints were retrieved, and then (2) the specific inclusion criteria, concerning participants, intervention, outcome measure, and study design, were applied for the evaluation of the remaining manuscripts. In cases of missing data, the authors were contacted to provide the original reports. Those cases that were deemed to be unclear were further discussed with the remaining authors of this review.

### 2.4. Data Extraction and Quality Assessment

The data extracted from each included study were the following: article identifiers (i.e., authors and year of publication); study design; sample size; stimulation parameters (i.e., protocol, target, intensity, number of sessions, and number of pulses per session) for the treatment group (TC) and, if applicable, for the control group (CG); any other non-surgical intervention concurrently applied with the stimulation; tools and measures used for assessing primary and secondary outcomes; and the effects of stimulation on PTSD symptoms’ severity and secondary outcomes. A standardized approach developed under the Effective Public Health Practice Project (EPHPP; [50]) was employed to critically evaluate the quality of and risk of bias in the reviewed studies as well as guarantee that they were reviewed with an equal scientific approach. Such critical appraisal tool was chosen since it consists of eight subdomains (i.e., selection bias, confounders, blinding, data collection methods, withdrawals and dropouts, intervention integrity, and analysis), providing a global rating of the methodological quality for each quantitative study included in this review, which could be (1) strong, (2) moderate, or (3) weak.

## 3. Results

**Figure 1:**
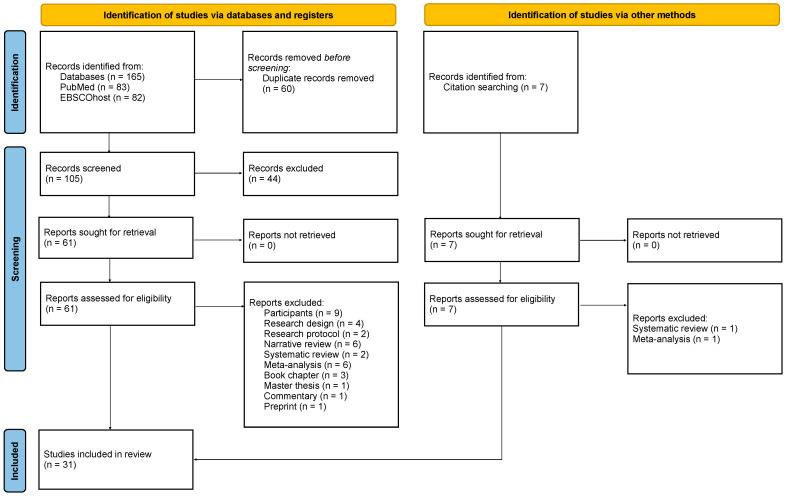
PRISMA flowchart diagram. From Page et al. [47]. For more information, visit http://www.prisma-statement.org/ (accessed on the 24 February 2024).

As shown in the PRISMA flow chart (Figure 1), 165 records were identified from searching across six databases. After duplicate removal, a total of 105 records were screened at the title and abstract levels. Manual searches from the articles’ references generated seven relevant records that were considered for full-text screening. A total of 68 reports (61 from the databases and 7 from the manual searches) were evaluated for full-text eligibility. Nine studies were excluded as they had been conducted on animals, healthy subjects, and patients with a primary diagnosis other than PTSD, i.e., specific phobia, persistent post-concussion syndrome, treatment-resistant depression, suicide behavior disorder, and traumatic brain injury syndrome. Three were correlational studies; one was a single-case study; and two were study protocols. Six narrative reviews, two systematic reviews, six meta-analyses, one Master-level dissertation, three book chapters, one commentary, and one preprint were also kept out of this work. Overall, 31 studies met the inclusion criteria for the data extraction process and, thus, were included in this systematic review [26,51,52,53,54,55,56,57,58,59,60,61,62,63,64,65,66,67,68,69,70,71,72,73,74,75,76,77,78,79,80].

### 3.1. Demographic Characteristics of the Sample

A total of 1260 subjects were included from all the studies discussed, reporting heterogenous traumatic experiences. Most of them were war veterans (55.6%), followed by clinical outpatients (32.8%), civilians (6.7%), and treatment-refractory patients (4.9%). The mean age of the sample was 40.55 years. A total of 66.9% of the sample was male, whereas 33.1% of subjects were females. Of the total subjects, 57.5% had a diagnosis of PTSD while 36.5% showed at least one comorbidity. The remaining 6% reported severe trauma-related symptoms but not a formal nosographical diagnosis of PTSD. The most frequent comorbid condition was MDD (88.6%), followed by SUD (17.9%), panic disorder (5.0%), generalized anxiety disorder (2.2%), specific phobia (1.7%), and obsessive compulsive disorder (1.3%). See Table 1 for more detailed information concerning each study.

### 3.2. Effects of Transcranial Magnetic Stimulation on Post-Traumatic Stress Disorder Symptoms’ Severity

Table 2 reports a summary of the characteristics of the included studies that made use of TMS (*n* = 26). The results are presented according to the type of repetitive TMS (rTMS) protocol applied in the studies. Repetitive TMS typically consist of identical stimuli spaced by an identical inter-stimulus interval (ISI), yet its effects depend on the stimulation frequency: at a low frequency (i.e., ≤1 Hz), rTMS depresses cortical excitability, whereas, at a high frequency (i.e., >5 Hz), cortical excitability is enhanced [81]. Theta-burst stimulation (TBS) involves bursts of high-frequency stimulation (i.e., three pulses at 50 Hz) repeated with an ISI of 200 ms (i.e., at 5 Hz). In the intermittent TBS (iTBS) protocol, bursts are usually delivered for 2 s and then repeated every 10 s (i.e., 2 s of TBS followed by a pause of 8 s). In the continuous TBS (cTBS) protocol, bursts are instead repeated for 40 s without any prolonged pause between them [82].

#### 3.2.1. Low-Frequency rTMS Protocols

Early evidence concerning the effects of TMS on PTSD symptomatology was produced by Grisaru et al. [57], who demonstrated that a single session of 0.3 Hz rTMS over the bilateral motor cortex resulted in a significant decrease in the avoidance and somatization sub-symptoms reported by a group of ten outpatients with PTSD. Such clinical improvements were observed after 24 h and 7 days from the treatment [57]. Rosenberg et al. [71] applied, instead, ten sessions of low- or intermediate-frequency rTMS targeting the left dlPFC of twelve subjects with combat-related PTSD, noticing that the core combat PTSD symptoms faced a modest decrease compared to the baseline, which remained significant at the 2-month follow-up. Notably, relevant differences in such clinical improvements were not found between the 1 Hz and 5 Hz rTMS groups [71]. Similar findings were obtained by subjecting twenty patients with PTSD to ten sessions of sham-controlled 1 Hz rTMS over the right dlPFC [77]. The intervention significantly ameliorated the overall PTSD symptoms’ severity reported by the active rTMS group compared to the sham rTMS group at both the 1-month and 2-month follow-ups. Nevertheless, no differences were detected between the two groups in treatment effectiveness across particular clusters of PTSD symptoms [77]. This evidence was partially refuted by a subsequent study conducted by Nam et al. [65] on sixteen patients with PTSD, highlighting that the administration of active 1 Hz rTMS over the right dlPFC versus a sham induced clinically relevant improvements not only in the overall PTSD symptoms’ severity but also in the CAPS avoidance and re-experiencing sub-scales. No significant differences were, however, detected in the hyperarousal sub-symptom of PTSD between the groups [65]. Conversely, the application of the same TMS protocol on twenty male subjects with combat-related PTSD led to a significant reduction in their hyperarousal symptoms following stimulation compared to the baseline [68]. Yet, no relevant differences were noted in the intrusion and avoidance PTSD sub-symptoms between the pre- and post-treatment conditions [68]. A stimulation protocol characterized by thirty-six sessions of intermediate pulse frequency (i.e., 5 Hz ) rTMS over the left dlPFC was also tested on ten veterans with PTSD, highlighting a significant improvement in their overall PTSD symptoms’ severity compared to the baseline [69]. These results were further corroborated in a prospective open trial conducted on a wider cohort of thirty-three outpatients with PTSD [26]. Zandvakili et al. [80] applied the same 5 Hz rTMS protocol to thirty-five individuals with PTSD and observed that stimulation was associated with significant baseline-to-endpoint improvements in their PTSD symptomatology.

#### 3.2.2. High-Frequency rTMS Protocols

The effectiveness of high-frequency rTMS on PTSD symptoms was demonstrated by Boggio et al. [53] by subjecting thirty patients to ten sessions of 20 Hz rTMS over the right or left dlPFC. A significant group-by-time interaction was detected, suggesting that both the right rTMS and the left rTMS groups exhibited a steeper decrease in their overall PTSD symptoms’ severity compared to the sham even at the 3-month follow-up [53]. Noteworthy, the right rTMS protocol induced a larger effect compared to the left rTMS; yet, the right rTMS induced greater reductions in the avoidance and hyperarousal sub-symptoms, and the left rTMS yielded a greater reduction in the re-experiencing sub-symptoms [53]. This evidence was confirmed by a subsequent retrospective study examining the PCL scores obtained by seventy-seven active-duty and post-active veterans with PTSD prior to and following the administration of thirty-one sessions of 10 Hz rTMS [78]. Compared to the baseline, the participants showed a significant decrease in their overall PTSD symptoms’ severity, which was also maintained at the 2-week follow-up [78]. Consistently, Madore et al. [63] conducted a multi-centric investigation involving one hundred forty-nine veterans with PTSD and observed a meaningful improvement in their clinical status. The majority of the sample underwent thirty-six sessions of 10 Hz rTMS over the left dlPFC, and, after treatment, they showed a significant reduction in their PCL scores compared to the baseline [63]. Noteworthily, Ahmadizadeh & Rezaei [52] investigated the efficacy of ten sessions of 20 Hz bilateral versus unilateral (right) rTMS applied over the dlPFC on PTSD symptomatology among fifty-eight male veterans. A significant main effect of time and a significant group-by-time interaction were obtained, suggesting that both the unilateral and bilateral rTMS groups attained a greater reduction in their overall PTSD symptoms’ severity compared to the sham at the end of the intervention [52]. Havign said that, the PCL scores of the bilateral versus unilateral rTMS groups were not significantly different upon completion of the treatment [52].

#### 3.2.3. Low- versus High-Frequency rTMS Protocols

The effects of low- versus high-frequency rTMS on PTSD symptomatology were first compared by Cohen et al. [55], who subjected twenty-four patients with PTSD to ten sessions of sham-controlled rTMS delivered at 1 Hz or 10 Hz over their right dlPFC. The authors observed a significant main effect on the TMS group and a significant group-by-time interaction, meaning that the overall PTSD symptoms’ severity diminished significantly in the 10 Hz rTMS group compared to the 1 Hz rTMS and sham groups [55]. Especially, 10 Hz rTMS was proven to be effective in lowering the re-experiencing, avoidance, and hyperarousal sub-symptoms of PTSD 24 days after the treatment [55]. This evidence was partially corroborated by Kozel et al. [61] through a clinical trial conducted on thirty-five veterans with PTSD aimed at testing the efficacy of thirty-six sessions of 1 Hz versus 10 Hz rTMS over the right dlPFC on PTSD-related symptoms. The authors highlighted that both the 1 Hz and 10 Hz rTMS groups showed clinically relevant improvements from the baseline to the end of the treatment in their overall PTSD symptoms’ severity, but no statistically significant differences were observed between the two groups at such timepoints [61]. Conversely, Leong et al. [62] subjected twenty-nine civilians with PTSD to ten sessions of active or sham rTMS delivered at 1 Hz or 10 Hz over the right dlPFC and demonstrated that the low-frequency protocol was superior to the high-frequency one. Specifically, the researchers obtained a significant group-by-time interaction, suggesting that the PTSD symptoms among those subjects receiving 1 Hz rTMS consistently improved compared to the sham group, whereas the symptoms of those receiving 10 Hz rTMS did not [62].

#### 3.2.4. TBS Protocols

Apart from high- and low-frequency rTMS protocols, theta-burst stimulation has also been proven to be effective in ameliorating PTSD symptomatology. Indeed, ten sessions of active versus sham iTBS over the right dlPFC of fifty veterans with PTSD produced a significant reduction in the overall PTSD symptoms’ severity among the active iTBS group compared to the sham iTBS group [54,70,75]. Also, twenty sessions of bilateral iTBS over the dlPFC effectively decreased PTSD-related symptoms among a smaller cohort consisting of eight Australian veterans [66]. In this study, the total CAPS scores demonstrated significant mean decrements between the pre- and post-treatment assessments, which were maintained at the 3-month follow-up [66]. Further, at the 3-month follow-up, the intrusion and hyperarousal PTSD sub-symptoms showed a slight shift back to the baseline, whereas avoidance and negative alterations in cognition and mood continued to improve [66]. Interestingly, Yuan et al. [79] compared the efficacy of high-frequency rTMS with that of iTBS on seventy-five civilians reporting highly severe PTSD symptoms (i.e., PCL scores ≥ 50). Intervention was characterized by fifteen sessions of sham-controlled 10 Hz rTMS or iTBS targeting the right dlPFC. A significant group-by-time interaction was observed, indicating that both the 10 Hz rTMS and iTBS groups showed faster and greater reductions in their overall PTSD symptoms’ severity compared to the sham at the end of the treatment and the 1-month follow-up [79]. Nevertheless, no significant group differences between the 10 Hz rTMS and iTBS groups were detected in terms of efficacy [79].

#### 3.2.5. Integrated Protocols

TMS was first combined with psychotherapy by Osuch et al. [67], who conducted a preliminary study on nine treatment-resistant patients with PTSD, unveiling a moderate improvement in their hyperarousal symptoms after twenty sessions of imaginal exposure therapy [83] administered during sham-controlled 1 Hz rTMS over the right PFC. Nonetheless, no statistically significant differences were found between the active and sham rTMS groups with respect to the avoidance and intrusion sub-symptoms of PTSD [67]. Similarly, Isserles et al. [58] subjected twenty-six patients affected by treatment-resistant PTSD to an integrated protocol characterized by twelve sessions of a custom script-driven imagery procedure followed by the administration of active or sham repetitive deep TMS (dTMS) over the medial prefrontal cortex (mPFC). After the treatment, the authors observed a significant group-by-time interaction, meaning that active dTMS coupled with traumatic exposure to imagery induced a sharp decrease in the CAPS intrusion component compared to the control groups [58]. For the avoidance and hyperarousal PTSD sub-symptoms, the interaction effect was not significant, but the pre-planned contrast highlighted significant improvements only in the active dTMS + exposure therapy group [58]. The same researchers applied an analogue treatment protocol to a wider cohort of one hundred twenty-five outpatients with PTSD and noticed that both the active dTMS + script-driven imagery and sham dTMS + script-driven imagery groups displayed a significant amelioration in their clinical conditions [59]. Nevertheless, the overall PTSD symptoms’ severity assessed 1 week after the treatment and at the 1-month follow-up was significantly lower in the sham dTMS + script-driven imagery group compared to the real dTMS + script-driven imagery group [59]. In a further preliminary investigation, Fryml et al. [56] highlighted that eight sessions of 10 Hz rTMS over the left or right dlPFC administered during 30 min prolonged exposure (PE, [83]) therapy resulted in a general non-significant trend towards an improvement from the baseline to the end of the treatment in both groups (i.e., sham rTMS + PE and real rTMS + PE). Consistently, Thierree et al. [73] subjected thirty-eight patients with PTSD to eight sessions of high- or low-frequency rTMS over the right dlPFC during a custom script-driven imagery procedure and observed a steep decrease in their overall PTSD symptoms’ severity from the baseline to the 3-month follow-up. However, no significant group-by-time interaction was obtained, suggesting that the 1 Hz and 10 Hz rTMS groups did not differ in terms of recovery [73]. Besides exposure-based approaches, TMS has also been paired with cognitive psychotherapy. Indeed, Kozel et al. [60] tested the efficacy of twelve sessions of sham-controlled 1 Hz rTMS over the right dlPFC combined with cognitive processing therapy (CPT, [84]) through a clinical trial conducted on one hundred and three veterans with combat-related PTSD. Both the active rTMS + CPT and sham rTMS + CPT groups showed a significant reduction in their overall PTSD symptoms’ severity from the baseline to the 1-month, 3-month, and 6-month follow-ups [60]. Notably, at all timepoints, the active rTMS + CPT group reported greater symptoms recovery compared to the sham rTMS + CPT group [60].

### 3.3. Effects of Transcranial Magnetic Stimulation on Anxiety and Depressive Symptoms’ Severity

Besides having a diagnosis of PTSD, many of the subjects included in this review reported anxiety and/or depressive symptoms. In some cases, they even had a comorbid nosographical diagnosis of MDD and/or anxiety disorders (see Table 1). The following paragraphs are dedicated to highlighting the effects of different TMS protocols on such a symptomatology among patients with PTSD.

#### 3.3.1. Low-Frequency rTMS Protocols

Grisaru et al. [57] unveiled that a single session of 0.3 Hz rTMS over the bilateral motor cortex lowered patients’ anxiety symptoms from the baseline to 28 days after the treatment but did not affect their depressive symptomatology. This evidence was refuted by Rosenberg et al. [71], who demonstrated that ten sessions of 1 Hz or 5 Hz rTMS over the left dlPFC resulted in a robust amelioration in anxiety and depressive symptoms compared to the baseline, which was largely sustained at the 2-month follow-up. However, no significant differences in mood recuperation were found between the 1 Hz and 5 Hz rTMS groups [71]. Watts et al. [77] consistently demonstrated that active 1 Hz rTMS over the right dlPFC versus a sham produced a significant reduction in depressive but not anxiety symptoms. Conversely, Oznur et al. [68] applied the same rTMS protocol on a single group of patients with PTSD, and they did not observe any relevant differences in anxiety and depressive symptoms’ severity between the pre- and post-treatment conditions. However, the application of 5 Hz rTMS over the left dlPFC efficiently lowered patients’ depressive symptoms’ severity from the baseline to the treatment’s endpoint [26,69,80].

#### 3.3.2. High-Frequency rTMS Protocols

Not only low-frequency rTMS but also high-frequency rTMS has been shown to alleviate anxiety and depressive symptoms considerably among subjects with PTSD. Indeed, ten sessions of 20 Hz rTMS over the right or left dlPFC have been shown to significantly lower anxiety and depressive symptoms, respectively [53]. These results were corroborated by Wilkes et al. [78], who asserted that thirty-one sessions of 10 Hz rTMS resulted in decreased BDI scores at the end of the treatment on average compared to the pre-treatment baseline scores. Similar findings were reported by Madore et al. [63], who demonstrated that thirty-six sessions of 10 Hz rTMS over the left dlPFC induced a significant reduction in veterans’ self-reported depressive symptoms’ severity compared to the baseline.

#### 3.3.3. Low- versus High-Frequency rTMS Protocols

Following ten sessions of sham-controlled 1 Hz or 10 Hz rTMS over the right dlPFC, Cohen et al. [55] obtained a significant main effect of rTMS and a significant treatment-by-time interaction, highlighting that anxiety and depressive symptoms decreased significantly in the 10 Hz rTMS group compared to the 1 Hz rTMS and sham groups both 10 days and 24 days after treatment [55]. Conversely, Kozel et al. [61] reported that, although both 1 Hz and 10 Hz rTMS over the right dlPFC produced relevant improvements in mood disturbances from the baseline to the end of the treatment, neither of the stimulation protocols was superior in terms of lowering depressive symptomatology [61]. This evidence was partially disconfirmed by Leong et al. [62], who detected a main effect of time but not a significant treatment-by-time interaction following the application of ten sessions of 1 Hz or 10 Hz rTMS over the right dlPFC with respect to anxiety- and depression-related manifestations. However, compared to the sham, the 10 Hz rTMS group reported marginal improvements in their depressive symptoms’ severity [62].

#### 3.3.4. TBS Protocols

Besides high- and low-frequency rTMS protocols, TBS has also appeared to be effective in lowering the severity of the depressive symptoms self-reported by patients with PTSD. Indeed, ten sessions of sham-controlled iTBS over the right dlPFC resulted in a meaningful reduction in depressive symptoms’ severity among the active iTBS group compared to the sham iTBS group at the end of the treatment [54,70,75]. Furthermore, twenty sessions of bilateral iTBS over the dlPFC consistently ameliorated veterans’ depressive symptoms in [66]. Indeed, a significant decrease in the HDRS scores was observed between the pre- and post-treatment assessments, which was modestly maintained at the 3-month follow-up [66].

#### 3.3.5. Integrated Protocols

A protocol of 1 Hz rTMS over the right PFC administered during twenty sessions of imaginal exposure therapy did not generate any relevant effect on the mood disturbances reported by a group of refractory patients with PTSD [67]. In line with these findings, twelve sessions of active dTMS over mPFC + script-driven imaginal trauma exposure did not produce a significant decrease in patients’ depressive symptoms’ severity compared to the sham in another study [58]. Considerable within-sessions reductions in the HDRS and BDI scores were, however, solely obtained among those subjects receiving active dTMS + exposure therapy [58]. This evidence was refuted by a multi-center study conducted by the same researchers, in which significant improvements in depressive symptomatology were detected in both the real and sham dTMS + script-driven imagery groups 1 week after the end of the treatment [59]. Consistently, Fryml et al. [56] observed a significant group-by-time interaction following the application of eight sessions of sham-controlled high-frequency rTMS + PE, thus suggesting that those patients receiving active 10 Hz rTMS over the left or right dlPFC along with PE had sharply lower depression scores relative to the baseline and compared with those subjects receiving the sham rTMS + PE treatment. In accordance with these findings, Thierree et al. [73] demonstrated that anxiety and depressive symptoms markedly decreased following trauma script exposure and 1 Hz or 10 Hz rTMS over the right dlPFC. Nonetheless, no significant group-by-time interaction was obtained, indicating that the 1 Hz and 10 Hz rTMS groups did not differ in terms of clinical improvement at the 3-month follow-up [73]. Not only exposure-based psychotherapy but also cognitive therapy augmentation seems to be effective in reducing depression among patients with PTSD. Indeed, Kozel et al. [60] demonstrated that active and sham 1 Hz rTMS protocols over the right dlPFC + CPT successfully ameliorated veterans’ depressive symptomatology. However, the active rTMS + CPT group was not superior to the sham rTMS + CPT group in terms of efficacy [60].

### 3.4. Effects of Transcranial Electrical Stimulation on Post-Traumatic Stress Disorder Symptoms’ Severity

Table 3 reports a summary of the characteristics of the included studies that utilized TES (*n* = 5). All of the investigations included made use of tDCS. No study employed tACS or tRNS. Contrary to TMS, TES does not directly induce neuronal firing but rather modulates cortical excitability by a polarity-dependent shift in the neuronal membrane’s potential [85]. The results are, therefore, presented according to the placement of the anode and cathode electrodes over the targeted brain region in the studies. Anodal tDCS is known to enhance cortical excitability, whereas cathodal tDCS has been shown to produce a reduction in cortical excitability [86].

#### 3.4.1. Anodal tDCS Protocols

Ahmadiazadeh et al. [51] tested forty outpatients with PTSD by applying ten sessions of sham-controlled anodal tDCS over the left dlPFC and demonstrated relevant ameliorations in their symptomatology. A significant main effect of time and a significant group-by-time interaction were obtained while considering the PCL main scores, suggesting that the overall PTSD symptoms’ severity diminished consistently in the active tDCS group compared to the sham tDCS group at both the post-test assessment and the 1-month follow-up [51]. A significant group-by-time interaction was also detected on the re-experiencing, negative alterations in cognition and mood, and hyperarousal sub-scales of the PCL, indicating a greater PTSD sub-symptoms’ reduction in the active tDCS group compared to the sham tDCS group at both the post-treatment evaluation and the 1-month follow-up [51]. However, no significant group-by-time Interaction was noticed regarding the avoidance sub-symptom of PTSD [51].

#### 3.4.2. Cathodal tDCS Protocols

Marcolin et al. [64] recruited eight civilians with PTSD who survived a nightclub fire and subjected them to ten sessions of cathodal tDCS over the right dlPFC. After treatment, the participants showed a significant reduction in their overall PTSD symptoms’ severity compared to the baseline, and such clinical improvements were maintained at the 1-month follow-up [64].

#### 3.4.3. Integrated tDCS Protocols

Six combat-related virtual reality (VR) exposure sessions combined with sham-controlled anodal tDCS over the vmPFC markedly ameliorated the clinical conditions of sixteen male veterans affected by warzone-related PTSD [74,76]. Specifically, both the active and sham groups displayed meaningful reductions in the overall PTSD symptoms’ severity after the treatment, which continued improving during the 1-month follow-up period [74]. These results were partially refuted by Smits et al. [72], who administered five sessions of sham-controlled anodal tDCS over the right inferior frontal gyrus (IFG) combined with 30 min inhibitory control training to ninety-six active-duty military personnel and post-active veterans with PTSD. Beside a significant reduction in the subjects’ overall PTSD symptoms’ severity over time, the active tDCS group versus the sham group did not significantly differ in terms of symptom level mitigation, except for a slightly greater decrease in the PCL scores in the active tDCS group versus the sham group due to the higher baseline PTSD symptoms’ levels in the first group [72]. Furthermore, even after the 3-month and 1-year follow-up periods, the treatment outcomes did not encounter substantive changes [72].

### 3.5. Effects of Transcranial Electrical Stimulation on Anxiety and Depressive Symptoms’ Severity

In addition to being diagnosed with PTSD, a substantial proportion of the subjects discussed in this review disclosed the presence of anxiety and/or depressive symptoms. Notably, in the TES studies, no individuals exhibited a concurrent nosological diagnosis of MDD and/or anxiety disorders (see Table 1). The ensuing paragraphs are dedicated to elucidating the impact of diverse TES protocols on the aforementioned clinical manifestations within a cohort of patients with PTSD.

#### 3.5.1. Anodal tDCS Protocols

Following the application of ten sessions of sham-controlled anodal tDCS over the left dlPFC, Ahmadizadeh et al. [51] unveiled a significant main effect of time and a significant group-by-time interaction while considering the BAI and BDI scores. This result indicated that anxiety and depressive symptoms dropped significantly in the active tDCS group compared to the sham tDCS group in both the post-treatment assessment and the 1-month follow-up [51].

#### 3.5.2. Cathodal tDCS Protocols

Marcolin et al. [64] highlighted that ten sessions of cathodal tDCS application over the right dlPFC resulted in a clinically relevant decrease in anxiety and depressive symptoms compared to the pre-treatment assessment levels, a result which remained significant until 3 months post intervention.

#### 3.5.3. Integrated tDCS Protocols

Five sessions of sham-controlled anodal tDCS over the right IFG combined with 30 min inhibitory control training did not consistently ameliorate anxiety- and depression-related manifestations among active-duty military personnel and post-active veterans with PTSD [72]. Accordingly, researchers observed a significant main effect of time but not a significant group-by-time interaction, thus indicating that the active tDCS group versus the sham group did not substantially differ in terms of anxiety and depressive symptom level mitigation at the end of the treatment [72].

## 4. Discussion

The primary aim of this systematic review was to summarize the current evidence concerning the therapeutic effects of NIBS among a clinical population affected by PTSD. Moreover, we focused on exploring the combined efficacy of NIBS and psychotherapy for PTSD. To achieve these goals, we manually screened scientific papers resulting from six databases and reported their characteristics based on the stimulation protocol applied in the studies.

With respect to rTMS, the efficacy of both high- and low-frequency stimulation protocols have been extensively tested over the last two decades, highlighting the fact that neuromodulation represents a suitable intervention for patients with refractory PTSD. Accordingly, in the studies we reviewed, both 1 Hz and 10 Hz rTMS significantly lowered core symptoms of this disorder, such as avoidance, hyperarousal, and the re-experiencing of traumatic events [53,55,65,68]. Also, the anxiety and depression that often occur alongside PTSD faced a significant reduction following high- or low-frequency rTMS application (e.g., [26,61,63,71,78]). Not only continuous protocols but also intermittent ones, such as iTBS, generated positive effects on PTSD-related symptoms, including avoidance and negative alterations in mood and cognition, as well as on depressive symptoms [54,66,70,75]. Although TMS consistently produced a relevant amelioration in patients’ clinical conditions, the protocols’ specifics, e.g., stimulation intensity, number of sessions, and number of pulses administered per session, were highly heterogenous, thus complicating the individuation of a precise protocol for the treatment of PTSD.

With regard to TES, both anodal and cathodal tDCS consistently decreased multiple core PTSD sub-symptoms [51,74]. After treatment, comorbid anxiety- and depression-related manifestations also encountered significant ameliorations [64]. However, once again, crucial tDCS protocol parameters, e.g., current intensity, current density, and number of sessions attended by participants, were markedly different among the studies. Compared to TMS, the number of TES investigations and their cumulative sample size were also significantly lower, thus highlighting the need to further explore the effectiveness of tDCS on PTSD and comorbid psychiatric disorders in future clinical trials.

**Figure 2:**
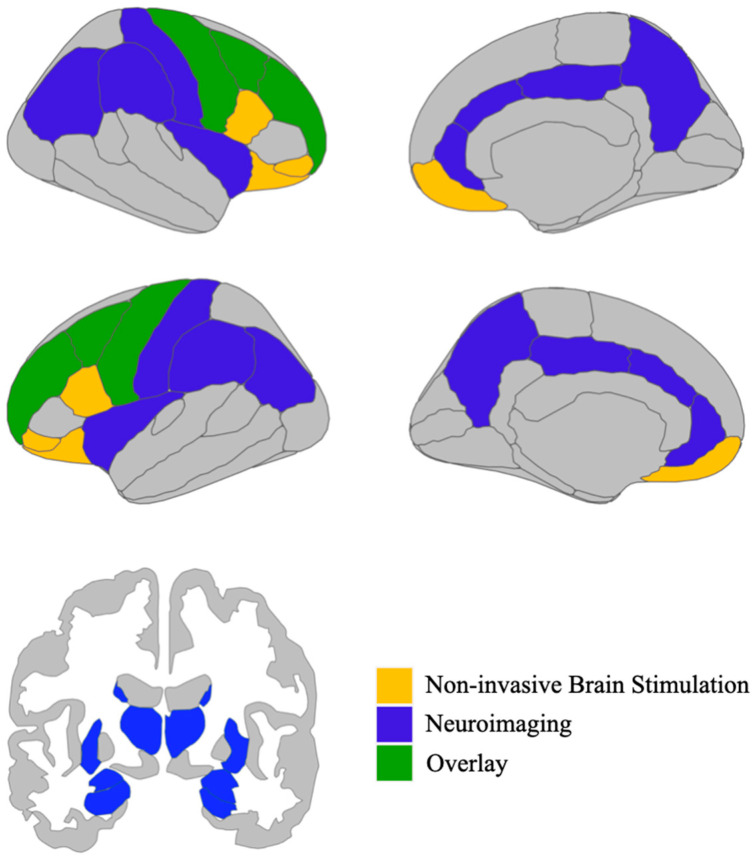
Overview of the brain regions enlisted in post-traumatic stress disorder according to neuroimaging studies (in blue), i.e., anterior cingulate cortex, posterior cingulate cortex, insula, precuneus, angular gyrus, supramarginal gyrus, postcentral gyrus, amygdala, hippocampus, putamen, caudate nucleus, and thalamus, depicted together with brain areas identified using non-invasive brain stimulation (in yellow), i.e., inferior frontal gyrus and orbitofrontal cortex. The the medial and dorsolateral prefrontal cortex as well as the precentral gyrus are implicated in post-traumatic stress disorder according to both non-invasive brain stimulation and neuroimaging investigations (in green). Image generation was facilitated through the utilization of the R (Version 4.3.2) package ggseg [96], which features data from the Desikan-Killiany cortical atlas [97] and the automatic segmentation of subcortical structures [98].

A common feature across the NIBS investigations included in this manuscript was the fact that neuromodulation was consistently applied over the dlPFC, a region known to play a crucial role in cognitive and emotional abnormalities characterizing PTSD [87,88]. Accordingly, neuroimaging studies demonstrated that patients suffering from PTSD showed a significant hypoactivation of the dlPFC and a consequent hyperactivation of the amygdala [89]. NIBS application over the PFC might, therefore, affect neuronal activity in the frontal-limbic network, enhancing top–down regulation over limbic structures highly implicated in the disorder, e.g., vmPFC ACC, hippocampus, and amygdala [26,90]. In line with this hypothesis, increasing PFC activation has been shown to result in a significant reduction in the emotional responses originating from the amygdala [91,92], evidence which can have impactful implications for the treatment of PTSD [93]. Besides the dlPFC, many other cortical and subcortical regions have emerged as being involved in PTSD, e.g., insula, precuneus, cingulate cortex, and angular and supramarginal gyrus (see Figure 2), whose anatomical location potentially allows for the employment of more advanced stimulation approaches in the future, aimed at counteracting neuronal dysregulation, e.g., high-definition transcranial direct-current stimulation (HD-tDCS). In this framework, NIBS represents a valuable tool to induce synaptic plasticity [94] and produce endurable changes in the activity and connectivity of large diffuse brain networks implicated in PTSD [95], thereby attenuating patients’ symptomatology in the long term.

Another major cue for reflection concerns the fact that both TMS and TES have been combined with psychotherapy, unveiling the capability of such integrated treatments to produce significant improvements in PTSD symptoms on multiple measures and lower comorbid anxiety and depression [56,58,59,67,72,73]. In this context, the putative effects of NIBS on neural plasticity are of great importance, as psychotherapy has been shown to enhance synaptic plasticity among patients with PTSD as well [99]. For instance, neuroimaging studies support that, after treatment, CBT induced an enhancement in the activation of prefrontal cortical subregions (e.g., inferior frontal gyrus, superior frontal gyrus, and middle frontal gyrus), the middle temporal gyrus, the parieto-temporal gyrus, and the hippocampus, accompanied by a decreased activation in the amygdala compared to the baseline [100,101]. Furthermore, these changes in neuronal activity were correlated with a reduction in PTSD symptoms’ severity [102,103,104]. Recent studies conducted on animal models of PTSD also highlighted that the application of alternating bilateral sensory stimulation (i.e., a visual EMDR protocol), produced a significant reduction in fear-related behavioral responses and yielded sustained increases in the activities of the superior colliculus and the thalamus compared to pre-treatment levels [105], likely attenuating the aftermath of traumatic memories. From this viewpoint, TES, whose range of action is not limited to cortical regions but is likely able to target subcortical areas as well, might be able to act directly on the same pathways involved in EMDR, such as the visual thalamus, altering its complex processing capabilities [106].

As both NIBS and psychotherapy appear to modulate the functioning of PTSD-related neural circuits while ameliorating subjects’ symptomatology, we speculate that an integrated treatment (i.e., NIBS + psychotherapy) might represent a promising approach for countering such mental disorder. Compared to psychotherapy alone, an integrated approach leads to a steeper reduction in PTSD as well as comorbid depressive symptoms’ severity, with PE therapy and CPT configuring among the most suitable candidates for such an intervention [58,60,67,73]. Nevertheless, no clear indications are provided regarding the exact order in which to apply NIBS and psychotherapy. In some cases, NIBS and psychotherapeutic treatment are administered simultaneously [56,67], whereas in some others NIBS application follows psychotherapy [58,59]. Of note, Kan et al. [44] suggested that both mono- and augmentation therapy with TMS yielded a significant positive effect on overall PTSD symptoms’ severity, but the effects were smaller for augmentation therapy compared with the controls, a phenomenon which might be due to the patients in the control groups benefiting from standalone psychopharmacological and/or psychotherapeutic treatment. Although drugs such as sertraline are also effective in ameliorating patients’ symptomatology when combined with PE therapy [107], NIBS has the advantage to focally modulate brain activity instead of inducing a widespread neuromodulatory effect and, typically, is not correlated with severe side effects [108].

Under this scenario, psychotherapy could gain advantage from NIBS application in terms of enhancing its efficacy and promoting faster and more durable decrements in PTSD symptoms [109,110]. Also, NIBS could benefit from psychotherapy as well, since the neuronal modifications induced by psychotherapeutic interventions would foster the synaptic changes caused by neurostimulation, making them more likely to take place and longer-lasting over time. In this framework, metaplasticity [111] and the non-linear effects of synaptic plasticity [112,113] might play a pivotal role, because they might produce complex and long-lasting changes in patients’ neuronal mechanisms, cognition, and behavior. It would be important, also, to gather a deeper insight in the structure and functioning of complex neural networks both in vitro and in vivo [114,115,116], especially with respect to changes induced by stress [25,30].

Concerning safety, the majority of patients enrolled in the included studies tolerated the stimulation without experiencing serious adverse effects. Indeed, side effects were generally mild or absent. Headache was the main side effect reported across the studies (e.g., [53,55]), followed by site pain during treatment [61] and increased intrusive thoughts [57]. Only one isolated case of suicidal ideation requiring hospitalization after one session of 1 Hz rTMS was reported [62]. These data underscore the overall safety and favorable tolerability of this kind of intervention.

Our systematic review suffers, however, from the following major limitations. The first one of these is the high heterogeneity of the studies due to the inclusion of a wide range of NIBS protocols and PTSD diagnostic criteria to ensure an updated and comprehensive overview of the field. However, this issue could be tackled through subsequent research endeavors concentrating exclusively on TES or directed towards the isolation of subtypes of PTSD for which NIBS techniques might prove more appropriate. Second, when reported, the types of traumas and time elapsed from the experience of traumatic events were highly dissimilar, which represent, instead, crucial factors for determining the different trajectories of PTSD symptoms and treatment response [117,118,119,120]. Third, the samples were mostly composed by men, and, in some cases, the sample size was small (e.g., [56,64,66]), limiting the generalizability of the findings. Furthermore, the inclusion of non-randomized and non-controlled studies lowered the quality of the evidence regarding the effect of NIBS on psychiatric symptomatology, which must, therefore, be taken with caution. Fourth, when NIBS was applied, several participants were currently under psychotherapeutic treatment and/or medication (e.g., [59,72,73]), which was, in some cases, not detailed enough to consent adequate systematization. Drugs, in particular, are known to affect significantly not only synaptic plasticity [121,122,123] but also the treatment response to NIBS interventions [124,125,126], possibly altering the results obtained from studies in which both strategies had been used. Fifth, many potentially relevant secondary outcomes were left out of this work to reduce complexity and permit a systematization focused on PTSD’s main symptoms. As an example, social functioning, cognitive capacities, and sleep quality were not considered, despite playing an important role in the course and prognosis of such a disorder [127,128,129]. Also, psychophysiological indexes were omitted, including skin conductance, heart rate variability, and serum hormones levels.

Besides these limitations, the above-mentioned findings have relevant implications for clinical practice. Indeed, clinicians could make use of NIBS in cases of treatment-resistant PTSD to boost the ameliorative effects of psychotherapy and thereby facilitate patients’ recovery. Especially, tDCS could be efficiently embedded in clinical practice due to its portability and reduced costs compared to TMS. Nonetheless, when selecting patients for NIBS therapy, clinicians must adhere to safety guidelines and recommendations concerning NIBS administration [19].

## 5. Conclusions

In conclusion, these preliminary findings indicate that NIBS consistently reduces the severity of both PTSD symptoms and comorbid anxiety and/or depressive symptoms among patients with a diagnosis of PTSD or highly severe PTSD symptoms. Nevertheless, larger randomized controlled trials with long-term follow-up assessments are necessary to corroborate these findings. If confirmed, clinicians could integrate the use of NIBS in their ordinary practice to treat patients who have not responded to first-line therapies or have experienced intolerable side effects from pharmacotherapy. Especially, the combination of NIBS with psychotherapeutic interventions such as PE or CPT seems to be a promising strategy and yet deserves to be ulteriorly tested in future clinical trials. In particular, investigations on the optimal timing and sequencing of NIBS and psychotherapy could represent a significant contribution for the development of an evidence-based integrated approach for the treatment of refractory PTSD. Moreover, the exploration of the effects of NIBS as a stand-alone therapy or in combination with psychotherapy on specific PTSD symptoms, such as avoidance, hyperarousal, re-experiencing, and negative alterations in cognition and mood, could provide useful insights into the use of NIBS in clinical practice. Further research could also explore whether NIBS-induced effects on PTSD also concern the higher-order cognitive processes of traumatized patients and if an eventual improvement correlates with PTSD symptoms’ severity. Among higher-order cognitive functions, delay discounting might represent a strategic candidate [130] since it is a transdiagnostic marker sensible to NIBS intervention that can be assessed through intertemporal choice tasks in both human subjects and animal models [131,132]. Another prompt for further investigations involves latent profile analysis [133], which would be a useful tool for identifying subgroups of patients with PTSD on which an integrated treatment could be suggested a priori or also turn out to be more effective in terms of symptoms’ mitigation.

## Figures and Tables

**Table 1:** Summary of the demographic characteristics of the samples from all the included studies.

ID	Mean Age (Standard Deviation)	Biological Sex	Population Type	Diagnosis	Comorbidity	Traumatic Event	Mean Years Elapsed since Trauma
Grisaru et al., 1998 [57]	40.50 (10.67)	7 males 3 females	Outpatients	PTSD	-	Accident, assault, combat	5.5
Rosenberg et al., 2002 [71]	54.80 (9.10)	12 males	Veterans	PTSD	MDD	Combat	28.9
Cohen et al., 2004 [55]	41.80 (6.93)	17 males 7 females	Outpatients	PTSD	-	Abuse, accident, assault, combat, death of a relative	5.5
Osuch et al., 2009 [67]	41.40 (12.30)	1 male 8 females	Treatment-refractory Patients	PTSD	-	-	22.3
Boggio et al., 2010 [53]	44.50 (4.40)	9 males 21 females	Outpatients	PTSD	-	Abuse, assault, combat, death of a relative, perceived threat of harm	3.9
Watts et al., 2012 [77]	55.90 (12.05)	18 males 2 females	Treatment-refractory Patients	PTSD	MDD, PD, OCD, SUD	Abuse, accident, assault, combat, multiple	39.8
Nam et al., 2013 [65]	34.31 (7.71)	6 males 10 females	Outpatients	PTSD	-	Accident, assault, domestic violence	3.3
Isserles et al., 2013 [58]	43.30 (10.93)	20 males 6 females	Treatment-refractory Patients	PTSD	-	-	15.8
Oznur et al., 2014 [68]	28.70 (3.30)	20 males	Veterans	PTSD	MDD	Combat	7.3
Philip et al., 2016 [69]	58.10 (13.9)	8 males 2 females	Veterans	PTSD	MDD	Combat	-
Kozel et al., 2018 [60]	-	103 males	Veterans	PTSD	-	Combat	-
Ahmadizadeh & Rezaei, 2018 [52]	50.45 (7.31)	58 males	Veterans	PTSD	-	Combat	25.8
Philip et al., 2018 [26]	51.30 (11.10)	10 males 13 females	Outpatients	PTSD	MDD	-	-
Fryml et al., 2019 [56]	28.50 (2.35)	7 males 1 female	Veterans	PTSD	-	Combat	-
Zandvakili et al., 2019 [80]	51.60 (10.30)	21 males 14 females	Veterans	PTSD	MDD	Combat	-
Philip et al., 2019 [70]	50.50 (12.50)	42 males 8 females	Veterans	PTSD	MDD, SUD	Combat	-
Kozel et al., 2019 [61]	38.50 (6.35)	25 males 10 females	Veterans	PTSD	-	Combat	9.3
Ahmadizadeh et al., 2019 [51]	43.75 (10.56)	14 males 26 females	Outpatients	PTSD	-	-	-
Marcolin et al., 2023 [64]	30.88 (7.74)	1 male 7 females	Civilians	PTSD	-	Perceived threat of harm, witnessing death, or serious injury	-
Van’t Wout-Frank et al., 2019 [74]	40.50 (8.80)	12 males	Veterans	PTSD	-	Combat	-
Leong et al., 2020 [62]	44.07 (10.93)	5 males 24 females	Outpatients	PTSD	MDD, GAD, SP, PD, OCD	Perceived threat of harm, sexual violence, witnessing death, serious injury, multiple	-
Nursey et al., 2020 [66]	37.52 (6.93)	7 males 1 female	Veterans	PTSD	-	Combat	-
Wilkes et al., 2020 [78]	39.50 (13.70)	41 males 36 females	Outpatients	PTSD	MDD	-	-
Van’t Wout-Frank et al., 2021 [75]	50.90 (12.20)	42 males 8 females	Veterans	PTSD	MDD, SUD	Combat	-
Isserles et al., 2021 [59]	44.25 (12.72)	42 males 83 females	Outpatients	PTSD	-	-	-
Bozzay et al., 2021 [54]	50.10 (12.30)	42 males 8 females	Veterans	PTSD	SUD	Combat	-
Thierreé et al., 2021 [73]	32.40 (10.55)	23 males 15 females	Outpatients	PTSD	-	Accident, assault, combat, perceived threat of harm	11.5
Smits et al., 2021 [72]	42.45 (10.00)	89 males 7 females	Veterans	PTSD	-	Combat	-
Van’t Wout-Frank & Philip, 2021 [76]	-	4 males	Veterans	PTSD	-	Combat	-
Madore et al., 2022 [63]	51.84 (14.11)	118 males 31 females	Veterans	PTSD	MDD	Combat	-
Yuan et al., 2023 [79]	34.71 (7.96)	20 males 55 females	Civilians	Highly Severe PTSD Symptoms	-	-	-

Note. PTSD = post-traumatic stress disorder; MDD = major depressive disorder; PD = panic disorder; SP = social phobia; GAD = generalized anxiety disorder; OCD = obsessive compulsive disorder; and SUD = substance use disorder.

**Table 2:** Summary of the studies making use of transcranial magnetic stimulation.

ID	Study Design	N	Brain Site TG	Protocol TG	Brain Site CG	Protocol CG	Augmentation	Clinical Measures	Effect	Assessment Timepoints	Study Quality
Grisaru et al., 1998 [57]	Open-label	10	Bilateral motor cortex	0.3 Hz rTMS, 100% RMT, 1 session, 30 pulses per session.	-	-	Antidepressants	IES, SCL-90	Decreased Avoidance, Anxiety, and Somatization.	Pre- and post-treatment, 1-week, and 1-month follow-ups	2
Rosenberg et al., 2002 [71]	Open-label	12	Left dlPFC	1 Hz or 5 Hz rTMS, 90% RMT, 10 sessions, 600 pulses per session.	-	-	Antidepressants	MISS, HDRS, POMS	Decreased Overall PTSD Symptoms’ Severity, Anxiety, and Depression.	Pre- and post-treatment, 1-month and 2-month follow-ups	1
Cohen et al., 2004 [55]	RCT	24	Right dlPFC	1 Hz or 10 Hz rTMS, 80% RMT, 10 sessions, 100 or 400 pulses per session.	Right dlPFC	Sham	Antidepressants, Anxiolytics, Mood Stabilizers, Antipsychotics	PCL, HARS, HDRS	Decreased Avoidance, Hyperarousal, Re-experiencing Anxiety, and Depression.	Pre- and post-treatment, 2-week follow-up	1
Osuch et al., 2009 [67]	Cross-over	9	Right dlPFC	1 Hz rTMS, 100% RMT, 20 sessions, 1800 pulses per session.	Right dlPFC	Sham	Exposure Therapy	CAPS, IES, HDRS	Decreased Hyperarousal.	Pre- and post-treatment	2
Boggio et al., 2010 [53]	RCT	30	Left or Right dlPFC	10 Hz rTMS, 80% RMT, 10 sessions, 1600 pulses per session.	Left or Right dlPFC	Sham	-	PCL, HARS, HDRS	Decreased Avoidance, Hyperarousal, Re-experiencing, Anxiety, and Depression.	Pre- and post-treatment, 2-week, 1-month, 2-month and 3-month follow-ups	1
Watts et al., 2012 [77]	RCT	20	Right dlPFC	1 Hz rTMS, 90% RMT, 10 sessions, 400 pulses per session.	Right dlPFC	Sham	-	CAPS, PCL, BDI, STAI	Decreased Overall PTSD Symptoms’ Severity and Depression.	Pre- and post-treatment, 1-month and 2-month follow-ups	1
Nam et al., 2013 [65]	RCT	16	Right dlPFC	1 Hz rTMS, 100% RMT, 15 sessions, 1200 pulses per session.	Right dlPFC	Sham	Antidepressants	CAPS	Decreased Avoidance and Re-experiencing.	Pre- and post-treatment, 1-month follow-up	1
Isserles et al., 2013 [58]	RCT	26	mPFC	dTMS, 120% RMT, 12 sessions, 1680 pulses per session.	mPFC	Sham	Antidepressants, Anxiolytics, Antipsychotics + Exposure Therapy	CAPS, PSS, BDI, HDRS	Decreased Intrusion.	Pre- and post-treatment, 2-week and 2-month follow-ups	1
Oznur et al., 2014 [68]	Open-label, retrospective	20	Right dlPFC	1 Hz rTMS, 80% RMT, 20 sessions, 600 pulses per session.	-	-	-	IES, BAI, BDI	Decreased Hyperarousal.	Pre- and post-treatment	2
Philip et al., 2016 [69]	Open-label	10	Left dlPFC	5 Hz rTMS, 120% RMT, 36 sessions, 3000 pulses per session.	-	-	-	PCL, QIDS	Decreased Overall PTSD Symptoms’ Severity and Depression.	Pre- and post-treatment	1
Kozel et al., 2018 [60]	RCT	103	Right dlPFC	1 Hz rTMS, 110% RMT, 12 sessions, 1800 pulses per session.	Right dlPFC	Sham	Cognitive Processing Therapy	CAPS, PCL, QIDS	Decreased Overall PTSD Symptoms’ Severity.	Pre- and post-treatment, 1-month, 3-month, and 6-month follow-ups	1
Ahmadizadeh & Rezaei, 2018 [52]	RCT	58	Bilateral or Right dlPFC	20 Hz rTMS, 100% RMT, 10 sessions, 2400 pulses per session.	Bilateral dlPFC	Sham	-	PCL	Decreased Overall PTSD Symptoms’ Severity.	Pre- and post-treatment	1
Philip et al., 2018 [26]	Open-label	33	Left dlPFC	5 Hz rTMS, 120% RMT, 36 sessions, 3000 pulses per session.	-	-	-	PCL, IDS	Decreased Overall PTSD Symptoms’ Severity and Depression.	Pre- and post-treatment	2
Fryml et al., 2019 [56]	RCT	8	Left or Right dlPFC	10 Hz rTMS, 120% RMT, 8 sessions, 6000 pulses per session.	Left or Right dlPFC	Sham	Antidepressants, Antipsychotics + Exposure Therapy	CAPS, PCL, HARS, HDRS	Decreased Depression.	Pre- and post-treatment	1
Kozel et al., 2019 [61]	Open-label	35	Right dlPFC	1 Hz or 10 Hz rTMS, 110% RMT, 36 sessions, 2400 pulses per session.	-	-	-	CAPS, PCL, MADRS, QIDS	Decreased Overall PTSD Symptoms’ Severity and Depression.	Pre- and post-treatment, 1-month and 3-month follow-ups	1
Philip et al., 2019 [70]	RCT	50	Right dlPFC	iTBS, 80% AMT, 10 sessions, 1800 pulses per session.	Right dlPFC	Sham	-	CAPS, PCL, IDS	Decreased Overall PTSD Symptoms’ Severity and Depression.	Pre- and post-treatment, 1-month follow-up	1
Zandvakili et al., 2019 [80]	Open-label	35	Left dlPFC	5 Hz rTMS, 120% RMT, 36 sessions, 3000 pulses per session.	-	-	-	PCL, IDS	Decreased Overall PTSD Symptoms’ Severity and Depression.	Pre- and post-treatment	2
Leong et al., 2020 [62]	RCT	29	Right dlPFC	1 Hz or 10 Hz rTMS, 120% RMT, 10 sessions, 2250 or 3000 pulses per session.	Right dlPFC	Sham	Antidepressants, Anxiolytics, Antipsychotics	CAPS, PCL, HDRS, QIDS, BAI, GAD-7	Decreased Overall PTSD Symptoms’ Severity and Depression.	Pre- and post-treatment, 3-month follow-up	1
Wilkes et al., 2020 [78]	Open-label, retrospective	77	-	10 Hz rTMS, 120% RMT, 31 sessions.	-	-	Antidepressants	PCL, BDI	Decreased Overall PTSD Symptoms’ Severity and Depression.	Pre- and post-treatment, 2-week follow-up	2
Nursey et al., 2020 [66]	Case series	8	Bilateral dlPFC	iTBS, 120% RMT, 20 sessions, 600 pulses per session.	-	-	Antidepressants, Anxiolytics, Mood Stabilizers, Antipsychotics	CAPS, HDRS	Decreased Avoidance, Negative Alterations in Cognition and Mood, and Depression.	Pre- and post-treatment, 3-month	1
Van’t Wout-Frank et al., 2021 [75]	RCT	50	Right dlPFC	iTBS, 80% AMT, 10 sessions, 1800 pulses per session.	Right dlPFC	Sham	-	PCL, IDS	Decreased Overall PTSD Symptoms’ Severity and Depression.	Pre- and post-treatment, 1-month follow-up	1
Bozzay et al., 2021 [54]	RCT	50	Right dlPFC	iTBS, 80% AMT, 10 sessions, 1800 pulses per session.	Right dlPFC	Sham	-	PCL, IDS	Decreased Overall PTSD Symptoms’ Severity and Depression.	Pre- and post-treatment, 1-month follow-up	1
Isserles et al., 2021 [59]	RCT	125	mPFC	dTMS, 120% RMT, 12 sessions, 1680 pulses per session.	mPFC	Sham	Antidepressants, Anxiolytics + Exposure Therapy	CAPS, MPSS, HDRS	Decreased Overall PTSD Symptoms’ Severity and Depression.	Pre- and post-treatment, 1-month follow-up	1
Thierreé et al., 2021 [73]	RCT	38	Right dlPFC	1 Hz or 10 Hz rTMS, 70% or 110% RMT, 8 sessions, 300 or 3000 pulses per session.	-	-	Antidepressants + Exposure Therapy	CAPS, PCL, HARS, HDRS	Decreased Overall PTSD Symptoms’ Severity, Anxiety, and Depression.	Pre-treatment, 1-month and 3-month follow-ups	1
Madore et al., 2022 [63]	Open-label, retrospective	149	Left dlPFC	10 Hz rTMS, 120% RMT, 36 sessions, 3000 pulses per session.	-	-	-	PCL, PHQ-9	Decreased Overall PTSD Symptoms’ Severity and Depression.	Pre- and post-treatment	1
Yuan et al., 2023 [79]	RCT	75	Right dlPFC	10 Hz rTMS or iTBS, 80% RMT, 15 sessions, 1800 pulses per session.	Right dlPFC	Sham	-	PCL	Decreased Overall PTSD Symptoms’ Severity.	Pre- and post-treatment, 1-month follow-up	1

Note. RCT = randomized controlled trial; dlPFC = dorsolateral prefrontal cortex; mPFC = medial prefrontal cortex; rTMS = repetitive transcranial magnetic stimulation; dTMS = deep transcranial magnetic stimulation; iTBS = intermittent theta-burst stimulation; AMT = active motor threshold; RMT = resting motor threshold; IES = Impact of Events Scale; SCL-90 = Symptoms Checklist-90; MISS = Mississippi Scale of Combat Severity; POMS = profile of mood states; PCL = PTSD Checklist; CAPS = Clinician-Administered PTSD Scale; PSS = PTSD Symptoms Scale; MPSS = Modified PTSD Symptom Scale; HDRS = Hamilton Depression Rating Scale; HARS = Hamilton Anxiety Rating Scale; BAI = Beck Anxiety Inventory; BDI = Beck Depression Inventory; STAI = State-Trait Anxiety Inventory; QIDS = Quick Inventory of Depressive Symptomatology; MADRS = Montgomery–Asberg Depression Rating Scale; IDS = Inventory of Depressive Symptomatology; GAD-7 = Generalized Anxiety Disorder-7; and PHQ-9 = Patient Health Questionnaire-9.

**Table 3:** Summary of the studies making use of transcranial direct-current stimulation.

ID	Study Design	N	Anodal/Cathodal Site TG	Protocol TG	Anodal/Cathodal Site CG	Protocol CG	Augmentation	Clinical Measures	Effect	Assessment Timepoints	Study Quality
Ahmadizadeh et al., 2019 [51]	RCT	40	Left dlPFC/Right dlPFC	2.0 mA, 0.57 A/m^2^, 10 sessions	Left dlPFC/Right dlPFC	Sham	-	PCL, BAI, BDI	Decreased Re-experiencing, Hyperarousal, Negative Alterations in Cognition and Mood, Depression, and Anxiety	Pre- and post-treatment, 1-month follow-up	1
Van’t Wout-Frank et al., 2019 [74]	Open-label	12	Left vmPFC/Right OC	2.0 mA, 0.22 A/m^2^, 6 sessions	-	-	Exposure Therapy	PCL	Decreased Overall PTSD Symptoms’ Severity	Pre- and post-treatment, 1-month follow-up	1
Van’t Wout-Frank & Philip, 2021 [76]	Pilot	4	Left vmPFC/Right OC	2.0 mA, 0.22 A/m^2^, 6 sessions	Left vmPFC/Right OC	Sham	Exposure Therapy	CAPS, PCL, QIDS	Decreased Overall PTSD Symptoms’ Severity	Pre- and post-treatment	2
Smits et al., 2021 [72]	RCT	96	Right IFG/Left OFC	1.25 mA, 0.04 A/m^2^, 5 sessions	Right IFG/Left OFC	Sham	Antidepressants, Anxiolytics, Mood Stabilizers, Antipsychotics, EMDR, and CBT + Inhibitory Control Training	PCL, STAI, BDI	-	Pre- and post-treatment, 3-month and 1-year follow-ups	1
Marcolin et al., 2023 [64]	Open-label	8	Left Deltoid Muscle	2.0 mA, 0.08 A/m^2^, 10 sessions	-	-	-	PCL, HARS, HDRS	Decreased Overall PTSD Symptoms’ Severity, Anxiety, and Depression	Pre- and post-treatment, 1-month and 3-month follow-ups	1

Note. RCT = randomized controlled trial; dlPFC = dorsolateral prefrontal cortex; vmPFC = ventromedial prefrontal cortex; IFG = inferior frontal gyrus; OFC = orbitofrontal cortex; OC = occipital cortex; EMDR = eye movement desensitization and reprocessing; CBT = cognitive–behavioral therapy; PCL = PTSD Checklist; CAPS = Clinician-Administered PTSD Scale; HDRS = Hamilton Depression Rating Scale; HARS = Hamilton Anxiety Rating Scale; BAI = Beck Anxiety Inventory; BDI = Beck Depression Inventory; STAI = State-Trait Anxiety Inventory; and QIDS = Quick Inventory of Depressive Symptomatology.

## Data Availability

Data sharing is not applicable to this article as no new data were created or analyzed in this study.
